# Phenosafranin-Based Colorimetric-Sensing Platform for Nitrite Detection Enabled by Griess Assay

**DOI:** 10.3390/s20051501

**Published:** 2020-03-09

**Authors:** Jingzhou Hou, Huixiang Wu, Xin Shen, Chao Zhang, Changjun Hou, Qiang He, Danqun Huo

**Affiliations:** 1 Key Laboratory of Eco-Environment of Three Gorges Region of Ministry of Education, College of Environment and Ecology, Chongqing University, Chongqing 400045, China; houjz@cqu.edu.cn; 2 Solid-state Fermentation Resource Utilization Key Laboratory of Sichuan Province, Yibin University, Yibin 644000, China; 2001106001@yibinu.edu.cn; 3 Key Laboratory for Biorheological Science and Technology of Ministry of Education, State and Local Joint Engineering Laboratory for Vascular Implants, Bioengineering College of Chongqing University, Chongqing 400044, China; cce.whx@gzhu.edu.cn (H.W.); 20151913064@cqu.edu.cn (X.S.); houcj@cqu.edu.cn (C.H.); 4 School of Chemistry and Chemical Engineering, Guangzhou Key Laboratory of Clean Energy and Materials, Guangzhou University, Guangzhou 510006, China; 5 Chongqing Key Laboratory of Bio-perception & Intelligent Information Processing, School of Microelectronics and Communication Engineering, Chongqing University, Chongqing 400044, China

**Keywords:** Griess assay, colorimetric, nitrite, phenosafranin

## Abstract

A facile and effective colorimetric-sensing platform based on the diazotization of phenosafranin for the detection of ${\mathrm{NO}}_{2}^{-}$ under acidic conditions using the Griess assay is presented. Diazotization of commercial phenosafranin produces a color change from purplish to blue, which enables colorimetric quantitative detection of ${\mathrm{NO}}_{2}^{-}$. Optimal detection conditions were obtained at a phenosafranin concentration of 0.25 mM, HCl concentration of 0.4 M, and reaction time of 20 min. Under the optimized detection conditions, an excellent linearity range from 0 to 20 μM was obtained with a detection limit of 0.22 μM. Favorable reproducibility and selectivity of the colorimetric sensing platform toward ${\mathrm{NO}}_{2}^{-}$ were also verified. In addition, testing spiked ham sausage, bacon, and sprouts samples demonstrated its excellent practicability. The presented colorimetric sensing platform is a promising candidate for the detection of ${\mathrm{NO}}_{2}^{-}$ in real applications.

## 1. Introduction

As one of the most important inorganic salt anions, nitrite (${\mathrm{NO}}_{2}^{-}$) plays significant roles in the fields of food, organic synthesis, pharmaceuticals, dyes, and agrochemicals [1,2,3,4,5]. However, the widespread use of nitrite poses a great threat to the environment due to its persistence in the soil and water [6,7]. In particular, in order to inhibit the propagation of toxic microorganisms in food and improve the color and flavor of meat products, nitrite has also been heavily used as an additive [8,9,10,11,12]. Extensive intake of nitrite can result in dysfunction of hemoglobin, digestive system cancer, and some other diseases [13,14,15,16,17]. In regards to the these problems, World Health Organization (WHO) and U.S. Environmental Protection Agency (EPA) set maximum contaminant levels (MCL) of nitrite in drinking water to 3.0 mg/L (65.1 μM) and 1.0 mg/L (21.7 μM), respectively [18]. Therefore, detection of nitrite is of great importance for environmental protection and food safety. 

Currently, numerous detection technologies have been developed for the nitrite analysis. For example, high performance liquid chromatography (HPLC) [19,20], ion chromatography (IC) [21,22], gas chromatography-mass spectrometry (GC-MS) [23,24], and other sensing platform [25] are widely employed for the detection of nitrite. Despite accuracy and sensitivity, the large-scaled equipment needed, complex samples pretreatment, and requirement of skilled personnel handicap their in-field applications. To address these issues, electrochemical sensors based on various nano-materials were fabricated [26,27,28]. Yet, the vulnerability of the electrode (ease of being poisoned) may influence the sensitivity and precision of detection. The reproducibility and selectivity could pose large challenges for real samples analysis based on electrochemical sensors [29,30]. Thus, the development of a robust and simple sensing platform for nitrite detection is urgently needed.

The colorimetric sensor is being examined by scientists and engineers in the field of nitrite discrimination due to its speed and ability to be visualized by naked eyes [31,32,33,34,35]. Among them, the catalytic spectrophotometric method was extensively investigated based on the reaction between an oxidizing agent (potassium bromate, potassium chlorate, potassium permanganate, and hydrogen peroxide) and some organic dyes in the presence of nitrite, which leads to the color change of dyes and quantitative detection of nitrite [36,37]. However, the time-consuming catalytic spectrophotometric method and the ease of disturbance by ${\mathrm{SO}}_{3}^{2-},{\;\mathrm{Br}}^{-},\;$ and $I^{-}$ hinder their widespread use in real application [38]. The Griess assay, as one of the most common alternatives to the colorimetric detection of nitrite, involves a diazo-coupling procedure under acidic conditions in the presence of nitrite and $\;-{\mathrm{NH}}_{2}$ on a certain chromophore, by which the color of sensing system changes and subsequently nitrite detection is achieved [18,39]. For example, some reported a spectrophotometric sensor based on diazotization of p-nitrophenol (Griess assay) to quantitatively detect nitrite [40,41]. Although a satisfactory detection goal can be obtained using a UV-visible spectrometer, the relatively small conjugation degree of the diazonium salt of p-nitrophenol (absorbance at 400 to 500 nm) is hard to visualize by the naked eye. Recently, Noor et al. [42] presented a new optosensor for visual quantitation of nitrite by physically immobilizing safranine O (SO) reagent onto a self-adhesive poly(n-butyl acrylate) (poly(nBA)) microspheres matrix. The large conjugate structure of SO (purplish) was diazotized through the Griess assay and formed a blue diazonium salt compound, which produces an obvious color change from purplish to blue and subsequently can be easily identified using the naked eye. The possible incomplete diazotization reaction resulted in a relatively high linear nitrite concentration ranging from 10 to 100 ppm (0.22 to 2.17 mM) in the solid phase, which does not meet the requirements set by the WHO (65.22 μM) and EPA (21.74 μM) [43].

**Figure 1:**
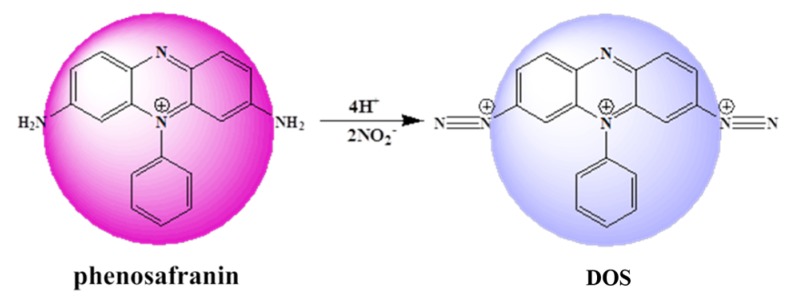
Scheme of proposed sensing platform.

To improve the practicality of the sensing platform for nitrite detection in the real world, we present a simple and effective colorimetric sensing platform based on the Griess assay. As shown in Figure 1, commercially cost-efficient phenosafranin (serving as the acting site and signal reporter) with large conjugated structure (purple) can be diazotized to form a blue diazonium salt (DOS) by nitrite under acidic conditions in the presence of nitrite in aqueous solution. Thus, qualitative and quantitative detection of nitrite was achieved according to the changes in absorbance of the UV-visible spectrum. In addition, the sensing platform was able to detect nitrite spiked into ham sausage, bacon, and sprouts with remarkable recovery. The results demonstrated that the sensing platform as presented displays excellent sensitivity and is robust against disturbance, and is a promising candidate for nitrite analysis in real samples.

## 2. Experiment

### 2.1. Reagents and Chemicals

Phenosafranin (PS, 80%) and NaHSO_3_ were purchased from Aladdin Chemistry (Shanghai, China). NaHCO_3_ was obtained from Chongqing Inorganic Chemical Reagent Plant. Disodium hydrogen phosphate (Na_2_HPO_4_) and sodium hydroxide (NaOH) were bought from Tianjin Chemical Reagent Co. Ltd. (Tianjin, China). A standard stock solution of ${\mathrm{NO}}_{2}^{-}$ was prepared using NaNO_2_ with ultrapure water. Other stock solutions of anions at 15 mM were prepared in ultrapure water with the corresponding salts with different anions of NaHCO_3_, Na_2_SO_4_, Na_2_SO_3_, Na_2_S_2_O_3_·5H_2_O, Na_2_S_2_O_5_, Na_2_CO_3_, Na_3_PO_4_·12H_2_O, Na_2_HPO_4_·12H_2_O, NaH_2_PO_4_, CH_3_COONa, C_6_H_5_O_7_Na_3_·2H_2_O, NaF, NaCl, NaBr, NaI, NaNO_3_, NH_4_NO_3_, NH_4_Cl, KCl, MgCl_2_, CuCl_2_·2H_2_O, MnCl_2_·4H_2_O, KSCN, FeCl_3_, and FeCl_2_·4H_2_O. All the above-mentioned chemicals were purchased from Chengdu Kelong Chemical Reagent Factory (Chengdu, China) except where indicated. All the above-mentioned reagents were of analytical purity unless otherwise stated. All aqueous solutions were prepared with deionized (DI) water (18.25 MΩ cm) from a Millipore water system.

### 2.2. Instruments

The UV-Vis absorption spectra obtained were recorded by a TU-1901 double-beam UV-vis spectrophotometer (Peking General Instrument Co. Ltd. Beijing, China). Fourier-transform infrared spectroscopy (FT-IR) with Spectrum GX Infrared and Microscopy System (PerkinElmer, USA) were used to confirm the chemical structure change of phenosafranin.

### 2.3. Preparation of Stock Solutions

To form 1 M aqueous stock solution for further use, 6.90 g of NaNO_2_ was dissolved in 100 mL deionized water. We prepared 1 M HCl aqueous solution by diluting 9 mL concentrated hydrochloric acid (36%–39%) into 100 mL deionized water. All the inorganic salts aqueous solutions were obtained by diluting them into 100 mL deionized water to form 0.1 μM stock solutions. All these aqueous solutions with certain concentrations were acquired by diluting corresponding stock solutions.

### 2.4. Preparation of Real Samples

Sprouts, bacon, and ham sausages bought from the local supermarket were selected as real samples for modeling the real world application of the proposed sensing platform. Pretreatment of these samples was performed according to the GB 5009.33-2016 (determination of nitrite and nitrate in food). Five grams of sample (sprouts, bacon, and ham sausage) was cut up and put into a 250 mL conical flask loaded with 10 mL deionized water to form a homogenate. After that, 12.5 mL saturated borax solution (50 g/L) and 150 mL hot deionized water (70 ℃) were added into the conical flask and incubated boiling water for 15 min. Then, the conical flask was taken out and cooled to room temperature. The extracting solution was transferred to 200 mL volumetric flask and added with 5 mL potassium ferricyanide aqueous solution (106 g/L). After shaking up, 5 mL zinc acetate solution (220 g/L) was added to precipitate the protein. Subsequently, deionized water was added into the volumetric flask to a total volume of 200 mL and allowed to stand for 30 min. Residuals were removed by filtering the stock solution was acquired. Finally, real samples were obtained after stock solution spiking with various concentrations of ${\mathrm{NO}}_{2}^{-}$.

### 2.5. Measurement Procedure

The analytical performance of phenosafranin toward ${\mathrm{NO}}_{2}^{-}$ was investigated at room temperature. Briefly, the phenosafranin solution (0.5 mL, 0.25 mM) was mixed with HCl aqueous solution (2 mL, 0.4 M) and loaded into a 5 mL centrifuge tube, followed by adding 1 mL ${\mathrm{NO}}_{2}^{-}$ in certain concentrations and incubating for 15 min. Finally, the UV-visible spectra of the mixture were recorded.

## 3. Results and Discussion

### 3.1. Sensing Mechanism

**Figure 2:**
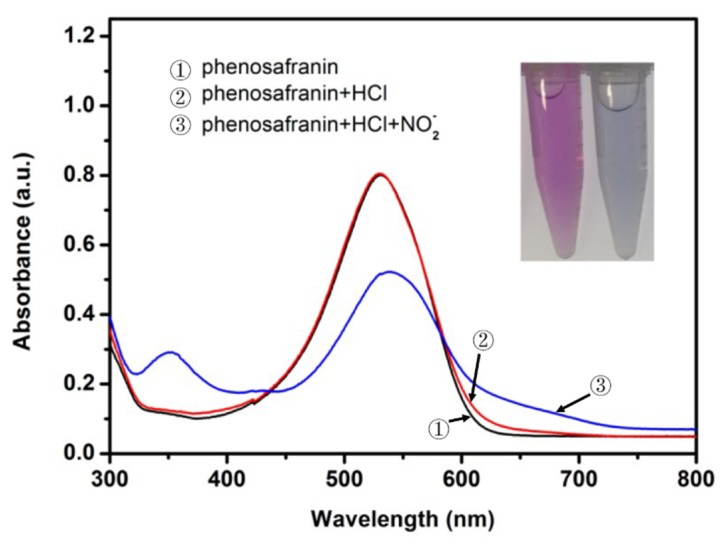
UV-visible spectra of phenosafranin (0.25 mM) before and after incubation with ${\mathrm{NO}}_{2}^{-}$ (10 μM) under acidic condition (HCl of 0.4 M) (inset: corresponding photo image with (right) and without (left)${\;\mathrm{NO}}_{2}^{-}$ ).

**Figure 3:**
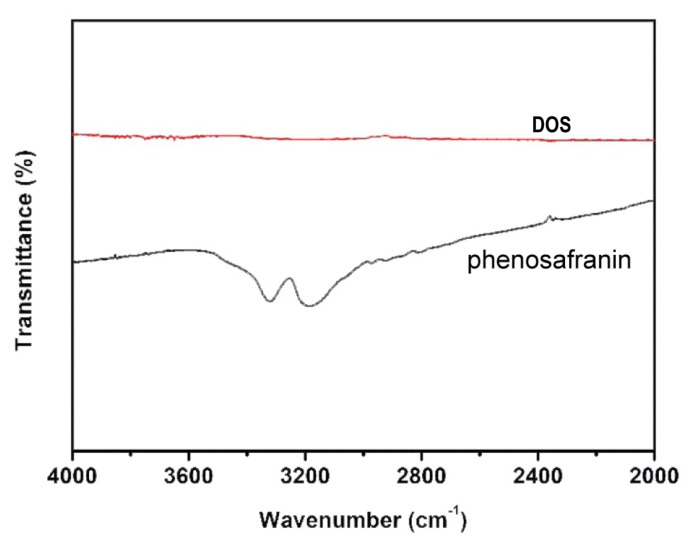
Fourier-transform infrared spectroscopy (FT-IR) spectra of phenosafranin and DOS.

The mechanism of the phenosafranin-based colorimetric-sensing platform is shown in Figure 1 and Figure 2. HCl was employed since the Griess assay occurs under acidic conditions. As shown in Figure 2, the UV-visible absorbance spectrum of phenosafranin was dominated by a single intense peak at 538 nm. No obvious change was observed for absorbance spectra of phenosafranin after incubation with HCl, indicating no influence of HCl on the color change of phenosafranin. Upon addition of ${\mathrm{NO}}_{2}^{-}$ at a concentration of 14 μM into phenosafranin solution with the presence of HCl, the strong absorbance peak at 538 nm receded and broadened, accompanied by a slight bathochromic shift (Figure 2), which can be ascribed to the diazotization of phenosafranin in agreement with a previous report [42]. As displayed in Figure 1, diazotization of $-{\mathrm{NH}}_{2}$ increases the conjugation degree of phenosafranin, resulting in the bathochromic shift of the absorbance peak. The color change of sensing system from red to blue can be clearly verified by the photo images in the inset of Figure 2. In order to demonstrate the chemical structure change, the DOS sample was first obtained by complete reaction between high concentration phenosafranin aqueous solutions with excess sodium nitrite under acidic conditions. Figure 3 shows the FT-IR spectra of phenosafranin and DOS in the wavenumber ranging from 2000 to 4000 cm^−1^. Two well-defined peaks at 3320.0 and 3185.0 cm^−1^ of phenosafranin are attributed to stretching vibration of amino group. After complete reaction with excessive ${\mathrm{NO}}_{2}^{-}$, the stretching vibration of amino group disappeared, suggesting the formation of diazonium salts DOS. Thus, colorimetric detection of ${\mathrm{NO}}_{2}^{-}$ based on phenosafranin under acidic conditions through the Griess assay could be achieved.

### 3.2. Optimization of Detection Conditions

**Figure 4:**
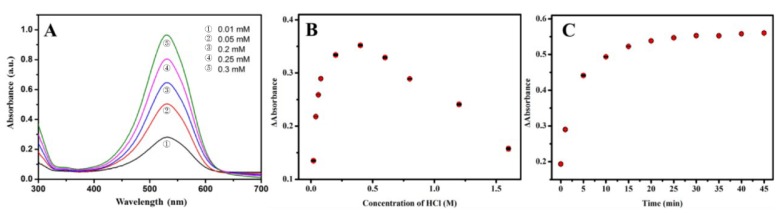
Optimization of concentrations of (**A**) phenosafranin (0.01, 0.05, 0.2, 0.25, 0.3 mM), (**B**) HCl (0.025, 0.05, 0.075, 0.1, 0.2, 0.4, 0.6, 0.8, 1.2, 1.6 M), and (**C**) reaction time (0, 1, 5, 10, 15, 20, 25, 30, 35, 40, 45 min).

In order to obtain an improved colorimetric response, the influence of concentrations of phenosafranin and HCl and incubation time were studied. As a sensing element and signal producer, a higher concentration of phenosafranin enables a more efficient diazotization reaction. However, quantitative detection is affected by the exorbitant concentration of phenosafranin due to the deviation from Lambert’s law. Additionally, a deep background disturbance influences the judgment on the color change of the sensing platform, extremely in the case of trace ${\mathrm{NO}}_{2}^{-}$ sensing. Therefore, appropriate concentration is important for the fabricated sensing platform. Figure 4A displays the absorbance spectra of phenosafranin at five concentrations. Taking these considerations into account, 0.25 mM phenosafranin was used for further studies. After optimization of phenosafranin concentration, the effect of concentration of HCl was also investigated. As shown in Figure 4B, the changes in the absorbance of phenosafranin (ΔA) at 532 nm increased with increasing HCl addition until it reached a maximum at a HCl concentration of 0.4 M and ${\mathrm{NO}}_{2}^{-}$ concentration of 14 μM, suggesting that HCl facilitates the diazotization of phenosafranin. At high HCl concentration (>0.4 M), ΔA decreased gradually with the increase in concentration of HCl, which may be contributed by the protonation of amidogen, being detrimental to diazotization [44]. Thus, we selected the HCl concentration of 0.4 M as optimal for conducting subsequent studies. Additionally, the influence of incubation time on ${\mathrm{NO}}_{2}^{-}$ detection even with large concentration (20 μM, to ensure the complete reaction time we can obtain) was researched and the test results are presented in Figure 4C. After a 10-minute incubation, an intense ΔA was obtained. After 20 min of reaction time, the ΔA leveled off, indicating that the reaction reached saturation. Typically, the reaction time of 20 min was chosen as the optimal time for further tests. Generally, we used the optimized phenosafranin concentration of 0.25 mM, HCl concentration of 0.4 M, and reaction time of 20 as favorable detection conditions for the investigation of the analytical performance of the sensing platform for ${\mathrm{NO}}_{2}^{-}$.

### 3.3. The Sensitivity of Colorimetric Sensing Platform

**Figure 5:**
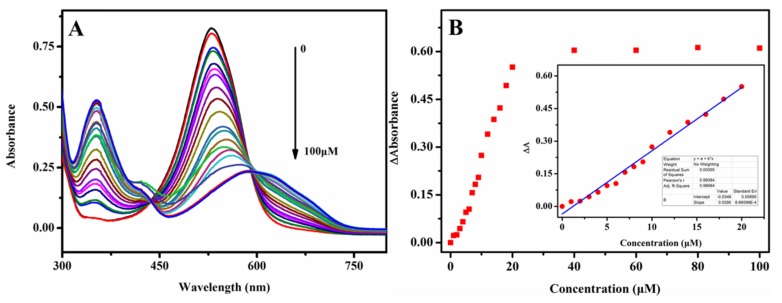
(**A**) UV spectra of phenosafranin (0.25 mM) at HCl concentration of 0.4 M versus ${\;\mathrm{NO}}_{2}^{-}$ with various concentrations from 0 to 100 μM and (**B**) corresponding ΔA at 532 nm of the sensing system at respective ${\;\mathrm{NO}}_{2}^{-}$ concentrations; (inset B: linear relationship between ΔA and ${\;\mathrm{NO}}_{2}^{-}$ concentrations ranging from 0 to 20 μM).

Under optimized detection condition, the sensitivity of the colorimetric sensor for ${\mathrm{NO}}_{2}^{-}$ was investigated and the test results are displayed in Figure 5. The UV-visible spectra, in the range from 300 to 750 nm of 0.25 mM phenosafranin in the presence of ${\mathrm{NO}}_{2}^{-}$ with various concentrations, are recorded in Figure 5A. Well-defined UV-visible absorbance peaks were obtained after incubation with ${\mathrm{NO}}_{2}^{-}$ in different concentrations. The absorbance peaks intensities decreased at 532 nm gradually with increasing concentrations ranging from 0 to 100 μM, accompanied by gradually increasing bathochromic shift. New stepped-up peaks at 350 nm arose with increasing ${\mathrm{NO}}_{2}^{-}$ concentrations. Figure 5B shows the ΔA at 532 nm with the addition of different concentrations of ${\mathrm{NO}}_{2}^{-}$. When increasing ${\mathrm{NO}}_{2}^{-}$ concentrations, ΔA increased accordingly. A well-defined linear relationship (R^2^ = 0.9977) between ΔA with ${\mathrm{NO}}_{2}^{-}$ ranging from 0 to 20 μM was obtained. The detection limit was calculated to be 0.22 μM (3S/N), which is much lower than the MCL of nitrite in drinking water set by the EPA and the WHO. Notably, satisfactory sensitivity and linear relationship range were acquired compared with the previously reported colorimetric sensing platform listed in Table 1, which are attributed to the large conjugated structure of phenosafranin and its highly reactive diazotization reaction. The low cost and ease of operation of this colorimetric sensing platform enable its widespread use. The test results demonstrated the potential feasibility of the sensing platform for the quantitative detection of ${\mathrm{NO}}_{2}^{-}$ within the requirements given by the EPA and WHO.

### 3.4. Interferences Study

**Figure 6:**
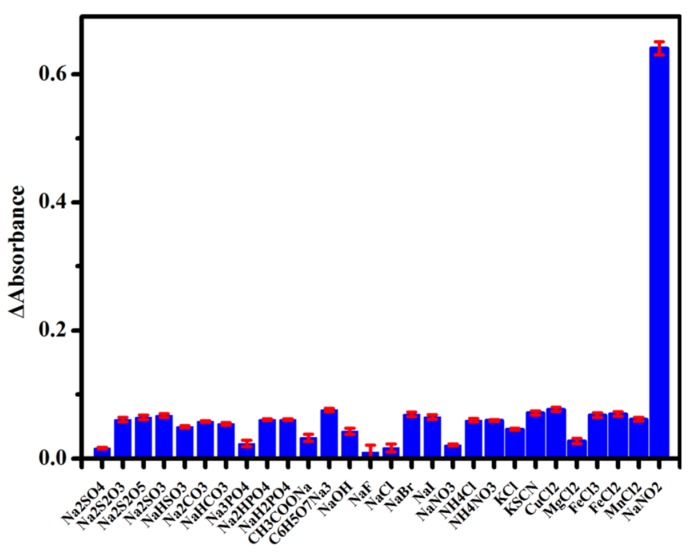
ΔA at 532 nm of the sensing platform for NaNO_2_ (35 μM) and other analytes (350 μM).

We conducted an interferences study to verify the selectivity of the colorimetric sensing platform. We selected 27 potential co-existing organic salts or alkali as interference chemicals to be tested. As shown in Figure 6, ΔA at 532 nm of the sensing platform after reaction with different substances (350 μM Na_2_SO_4_, NaHSO_3_, Na_2_S_2_O_3_, Na_2_SO_3_, Na_2_S_2_O_5_, Na_2_CO_3_, NaHCO_3_, Na_3_PO_4_, Na_2_HPO_4_, NaH_2_PO_4_, CH_3_COON_a_, C_6_H_5_O_7_Na_3_, NaOH, NaF, NaCl, NaBr, NaI, NaNO_3_, NH_4_Cl, NH_4_NO_3_, KCl, KSCN, CuCl_2_, MgCl_2_, FeCl_2_, FeCl_3_, and MnCl_2_) under optimal conditions were recorded, suggesting little influence on the sensing platform from these interferences. In contrast, ΔA (absorbance change) at 532 nm of the as-presented sensing platform after incubation with 35 μM of NaNO_2_ under the same detection conditions significantly increased, indicating a good selectivity of this sensing platform toward ${\mathrm{NO}}_{2}^{-}$. The selectivity of the sensing platform was assigned to the diazotization reaction of phenosafranin with ${\mathrm{NO}}_{2}^{-}$. The results indicated that the colorimetric sensing platform presented here has a good anti-disturbance ability.

### 3.5. Reproducibility Study

**Figure 7:**
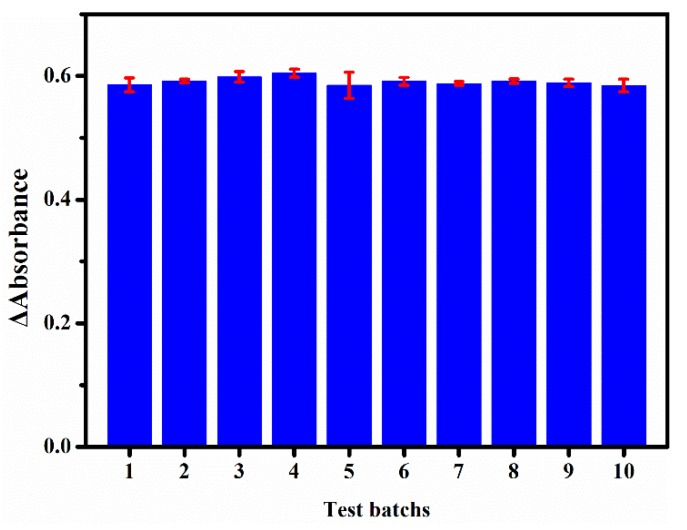
ΔA at 532 nm for the sensing platform for 10 batches of tests (35 μM of NaNO_2_) at different times.

To evaluate the stability, we recorded ΔA at 532 nm for the sensing platform upon incubation with 35 μM NaNO_2_ under the optimized detection conditions. Ten batches with six replicates for one batch were tested and the results are shown in Figure 7. An excellent reproducibility (relative standard deviation (RSD) = 1.59%) of the colorimetric sensing platform was obtained. 

### 3.6. The Practicability of the Colorimetric Sensing Platform

Various concentrations of ${\mathrm{NO}}_{2}^{-}$ spiked ham sausage, bacon, and sprouts samples (real sample pretreatment procedure was described in Preparation of real samples) were tested to evaluate the practicability of the colorimetric sensing platform. The ΔA at 532 nm of ham sausage, bacon, and sprouts samples spiked with ${\mathrm{NO}}_{2}^{-}$ were recoded to calculate the recoveries (Table 2). The detection results matched well with the accurate concentration for nearly all cases spiked with 0, 10, and 20 μM of ${\mathrm{NO}}_{2}^{-}$, demonstrating the good practicability of the colorimetric sensing platform. The recoveries acquired were calculated to range from 94.63% to 109.94%, with favorable reproducibility (RSD < 2%). These results suggested that the colorimetric sensing platform displays the potential for trace $NO_{2}^{-}$ detection in real applications.

## 4. Conclusions

We presented a simple and effective colorimetric sensing platform based on the diazotization of phenosafranin for the detection of ${\mathrm{NO}}_{2}^{-}$ under acidic conditions using the Griess assay. Commercial phenosafranin served as both acting site and signal producer. The color of phenosafranin changed from purplish to blue upon addition of ${\mathrm{NO}}_{2}^{-}$, enabling colorimetric sensing to achieve quantitative detection of ${\mathrm{NO}}_{2}^{-}$. A satisfactory sensitivity and favorable linearity were obtained under the optimal detection conditions. Interferences and reproducibility studies were conducted verify the selectivity and stability of the sensing platform. The testing of spiked real samples (ham sausage, bacon, and sprouts) confirmed its excellent practicability. All the results suggested the presented colorimetric sensing platform is a promising strategy for ${\mathrm{NO}}_{2}^{-}$ analysis in real applications.

## Figures and Tables

**Table 1:** Comparison of the analytical performance of previously reported colorimetric sensors with this work.

Reagents	Linearity Ranges (μM)	Detection Limits (μM)	Reference
azo-BODIPY	0–50	0.5	[8]
Ruthenium complexes	1–840	0.39	[32]
4-ATP/AuNPs	1–25	1	[33]
Griess reagent	1.6–21.74	0.86	[34]
safranine O (SO)	10–100 ppm	3 ppm	[41]
phenosafranin	0.1–20	0.22	this work

**Table 2:** Detection of $\;{\mathrm{NO}}_{2}^{-}$ in spiked ham sausage, bacon, and sprouts samples.

Samples	Added (μM)	Found (μM)	RSD (%)	Recoveries (%)
ham sausage	0	1.68	/	/
	10	12.6	1.5	108.97
	20	20.6	1.3	94.63
bacon	0	1.56	/	/
	10	11.9	1.4	103.74
	20	20.8	1.2	96.21
sprouts	0	1.93	/	/
	10	12.9	1.8	109.94
	20	21.1	1.3	95.91
